# Differences in DNA methylation profiles by histologic subtype of paediatric germ cell tumours: a report from the Children’s Oncology Group

**DOI:** 10.1038/s41416-018-0277-5

**Published:** 2018-10-05

**Authors:** Lindsay A. Williams, Lauren Mills, Anthony J. Hooten, Erica Langer, Michelle Roesler, A. Lindsay Frazier, Mark Krailo, Heather H. Nelson, Jessica Bestrashniy, James F. Amatruda, Jenny N. Poynter

**Affiliations:** 1 Division of Epidemiology & Clinical Research, Department of Pediatrics, University of Minnesota, Minneapolis, MN USA; 2 Minnesota Supercomputing Institute, University of Minnesota, Minneapolis, MN USA; 3 Dana-Farber/Boston Children’s Cancer and Blood Disorders Center, Boston, MA USA; 4 Department of Preventative Medicine, University of Southern California, Los Angeles, CA USA; 5 Division of Epidemiology and Community Health, University of Minnesota School of Public Health, Minneapolis, MN USA; 6 Masonic Cancer Center, University of Minnesota, Minneapolis, MN USA; 7 Departments of Pediatrics, Molecular Biology and Internal Medicine, University of Texas Southwestern Medical Center, Dallas, TX USA

**Keywords:** Cell biology, Germ cell tumours

## Abstract

**Background:**

Abnormal DNA methylation may be important in germ cell tumour (GCT) aetiology, as germ cells undergo complete epigenetic reprogramming during development. GCTs show differences in global and promoter methylation patterns by histologic subtype. We conducted an epigenome-wide study to identify methylation differences by GCT histology.

**Methods:**

Using the Illumina HumanMethylation450K array we measured methylation in 154 paediatric GCTs (21 germinomas/seminomas/dysgerminoma, 70 yolk sac tumours [YST], 9 teratomas, and 54 mixed histology tumours). We identified differentially methylated regions (DMRs) between GCT histologies by comparing methylation beta values.

**Results:**

We identified 8,481 DMRs (FWER < 0.05). Unsupervised hierarchical clustering of individual probes within DMRs resulted in four high level clusters closely corresponding to tumour histology. Clusters corresponding to age, location, sex and FFPE status were not observed within these DMRs. Germinomas displayed lower levels of methylation across the DMRs relative to the other histologic subtypes. Pathway analysis on the top 10% of genes with differential methylation in germinomas/seminomas/dysgerminoma compared to YST suggested angiogenesis and immune cell-related pathways displayed decreased methylation in germinomas/seminomas/dysgerminoma relative to YST.

**Conclusions:**

Paediatric GCT histologies have differential methylation patterns. The genes that are differentially methylated may provide insights into GCT aetiology including the timing of GCT initiation.

## Introduction

Germ cell tumours (GCTs), arising from totipotent primordial germ cells (PGC), are histologically heterogeneous tumours found in males and females in both gonadal and extragonadal locations. The main histologic subtypes of GCTs in the paediatric and adolescent population include: germinoma arising in the brain, yolk sac tumour (YST), seminoma/dysgerminoma, and teratomas (mature and immature).^1^ Tumours of mixed histology, that includes embryonal carcinoma and/or choriocarcinoma are also common.^1^ The predominant histologic subtype and tumour location differ by sex and age.^2^ GCTs are more frequently diagnosed in males when considering children aged 0–19 years.^3,4^ Among males < 5 years of age, tumours are equally distributed among extragonadal locations and the testes.^3^ In males aged < 5 years, the most common histology is YST and/or teratomas; conversely, GCTs in adolescent males are most often of mixed histologic subtypes and/or seminoma and are more frequently found in the testes.^2,3,5–7^ In contrast, for females, tumours are predominantly extragonadal prior to the age of 5 and then switch to a gonadal predominance throughout adolescence. As with males, the most common histology among females < 5 years of age is generally YST and/or teratomas, whereas in adolescence, the most common histologic subtypes are dysgerminoma and teratoma.^2^ Extragonadal tumours are more common in the paediatric age range compared to adolescent or young adult tumours, and are thought to arise from abnormal migration of PGCs during embryogenesis.^8,9^ As germ cells undergo extensive epigenetic reprogramming during the embryonic and early developmental periods,^6,10–12^ understanding differences in methylation patterns between histologic subtypes may shed light on aetiologic variation by histology as we work toward identifying important windows of exposure.

While strong risk factors for GCTs have yet to be identified, methylation changes in PGCs may be a biologic mechanism of tumour initiation and growth^11^ and may be influenced by environmental exposures during critical windows of development. Germ cells undergo two cycles of demethylation and reestablishment of methylation as part of normal development during embryogenesis and development, which may present relevant windows of susceptibility whereby environmental exposures to toxicants or stress may have lasting impacts on DNA methylation.^12^ Anomalous DNA methylation contributes to carcinogenesis by regulating gene expression and may be especially relevant for GCT development due to the extensive epigenetic reprogramming that occurs during embryogenesis and development.^12,13^ In general, paediatric tumours are less likely than adult cancers to result from the accumulation of mutations or DNA damage possibly due to the short latency period from birth to cancer diagnosis.^6,14^ Therefore, other mechanisms such as aberrant DNA methylation in GCT development may play an essential role in GCT initiation and progression, particularly among paediatric GCTs.

The histologic subtypes of GCTs display a gradient of global methylation with seminomas nearly lacking global methylation and less differentiated tumours (e.g., embryonal carcinoma) experiencing lower levels of global methylation relative to more differentiated tumours (e.g., teratoma).^15–23^ Differences in promoter methylation and expression have also been reported by GCT histology, particularly within key germ cell developmental signalling pathways including the BMP/TGF beta pathways.^7,19^ Differences in methylation patterns at imprinted loci between histologic subtypes of paediatric GCTs have also been reported.^20^ To further characterise differences in methylation between the histologic subtypes of paediatric GCTs, we conducted an epigenome-wide study to examine differences in methylation by histologic subtype among a histologically diverse set of 154 paediatric tumours including germinomas/seminomas/dysgerminomas, teratomas, yolk sac and mixed histologic subtype from gonadal, extragonadal and intracranial locations.

## Methods

### Samples

Tumour specimens were available for 154 paediatric GCTs (21 germinomas/seminoma/dysgerminoma, 54 mixed, 9 teratomas, 70 yolk sac tumours [YST]) collected from three sources (University of Texas Southwestern Medical Centre, Dallas, TX USA; Children’s Oncology Group; Boston Children’s Hospital, Boston, MA USA). Patient and tumour characteristic data were abstracted from medical records and pathology reports including patient age (0–10, 11–19 years), patient sex (male, female, gonadal dysgenesis, unknown), tumour histology (germinomas/seminoma/dysgerminoma, teratoma, yolk sac [pure yolk sac or teratoma and yolk sac histology], mixed/other [choriocarcinoma and embryonal carcinoma]) and tumour location (intracranial, extragonadal, gonadal [testis, ovary]). Informed written consent was obtained prior to sample collection at the treating institution. All study protocols were approved by the University of Minnesota Institutional Review Board.

### Methylation analysis

Prior to methylation analysis, 500 ng genomic DNA was treated with sodium bisulphite using the EZ DNA Methylation Kit (Zymo Research, Orange, CA) according to the manufacturer’s protocol. Genome-wide methylation analysis was performed using the Infinium HumanMethylation450 BeadChip array (Illumina, San Diego, CA) at the University of Minnesota Genomics Centre following Illumina’s standard protocol. For FFPE samples, we used the Infinium FFPE DNA Restore Kit (Illumina, Inc.) to repair DNA prior to array methylation analysis. All DNA samples were assessed for quality prior to analysis and duplicates were included for 19 samples to control for chip variation. The methylation status of a specific CpG site was calculated as the variable *β*, which is the ratio of the fluorescent signal from a locus specific probe to the methylated allele to the sum of the fluorescent signals of locus specific probes for both methylated and unmethylated alleles.^24^ These values range from 0 (unmethylated) to 1 (fully methylated).

### Quality control and determination of methylation levels

**Fig. 1:**
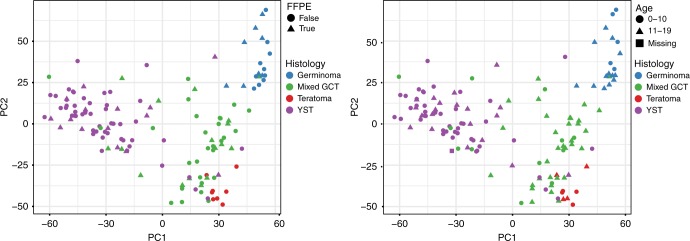
Principal component analysis was performed using quantile normalised methylation values from all probes for all samples with histology and FFPE status that had arrays after removing duplicate and control samples. **a** Point colours indicate tumour histology while shape indicates FFPE status. **b** Point colours indicate tumour histology while shape indicates patient age. Germinoma includes germinoma, seminoma and dysgerminoma

Array quality control and analysis was performed in R (v3.4.1) using the minfi package (v1.18.2).^25,26^ Arrays were checked for incomplete bisulphite conversion and overall beta value distributions. Outlier arrays were removed from the analysis. There was no evidence of batch effects similar to what has been reported elsewhere.^27^ Probes with known SNPs were removed from the study and the methylation status of the X and Y chromosome in each sample was checked against the reported sex to look for mis-labelling or other array errors. FFPE (*n* = 50, 32.5%) and frozen (*n* = 104, 67.5%) tumour samples were not distinctly different from one another (Fig. 1a) as has been observed between tumour samples in a recent validation study.^28^ Beta values were calculated for each probe using quantile normalisation.

### Principal component analysis (PCA)

PCA was performed using quantile normalised methylation (beta) values for or all samples with known histology and FFPE status via the prcomp function in R (v3.4.1) using the covariance matrix (scale = FALSE).

### DMR analysis

Differentially methylated regions (DMRs) were identified using bumphunter from the minfi package and quantile normalised beta values. DMRs were identified using generalised linear models and a smoothing technique as part of the bumphunter algorithm. The use of DMRs as the informative measure of differential methylation allowed us to capture groups of CpGs within the same region that possess the same methylation patterns while also capturing individual CpG loci that are differentially methylated. Only arrays with information for FFPE and histology were used during DMR tests. Family-wise error rates (FWER) for the DMR tests were computed using the bootstrap null method (*B* = 200). The DMR cutoff was 0.2 or 20%, as used in other methylation analyses and validation studies.^20,29–31^ The closest gene was identified for each DMR using bedtools closestBed and hg19 RefSeq gene annotations.

### Clustering

The heatmap was created in R (v3.4.1) using the pheatmap package on the quantile normalised methylation (beta) values. Hierarchal clustering using complete Euclidean distance was used to cluster samples (columns) and probe/DMR methylation values (rows) into similar groups. Samples with FFPE status and histology were included in the DMR calculations (18 germinomas/seminomas/dysgerminoma, 45 mixed tumours, 8 teratoma and 65 YST).

### Ingenuity pathway analysis

The top 10% of genes based on differences in methylation values that showed increased methylation in germinomas/seminomas/dysgerminoma relative to YST (*n* = 300) and the top 10% (*n* = 550) showing increased methylation in YST over germinomas/seminomas/dysgerminoma were entered into IPA to identify pathways of interest. The 10% of genes with differential methylation in germinomas/seminomas/dysgerminoma vs. YST were selected as a way to reduce the number of genes included in the pathway analysis, which is generally recommended to be conducted using a few hundred genes.^32^ The difference in methylation beta value between the two histologic subtypes was entered into IPA and pathways were identified using the IPA Knowledge Base gene list. Pathways with only one gene listed were omitted.

### Ratio of DMRs to total probes

The total number of probes with an absolute value for the differences in methylation beta value >0.2 between germinomas/seminomas/dysgerminoma compared to YST was calculated. If

*β*_germinomas/seminomas/dysgerminoma_ – *β*_YST_ was less than −0.2 these probes were considered to have increased methylation in YST relative to germinomas/seminomas/dysgerminoma. If *β*_germinomas/seminomas/dysgerminoma_ – *β*_YST_ was >0.2 these probes were considered to have increased methylation in germinomas/seminomas/dysgerminoma compared to YST. The ratio of DMRs to total probes was calculated by taking the number of probes with the appropriate difference in beta value divided by the total number of probes per chromosome.

### Identification of DMRs with extreme differences in methylation by histology

We also identified DMRs with extreme differences in methylation in germinomas/seminomas/dysgerminoma compared with YST as these may be genes of aetiologic importance. For this analysis, we classified *β* < 0.3 as hypomethylated and *β* > 0.7 as hypermethylated. We then selected DMRs that were hypomethylated in germinomas/seminomas/dysgerminoma and hypermethylated in YST and DMRs with *β* > 0.7 (hypermethylated) in germinomas/seminomas/dysgerminoma and *β* < 0.3 (hypomethylated) in YST. The differential *β* was calculated as *β*_germinomas/seminomas/dysgerminoma –_ *β*_YST._ We also determined which DMRs had similar methylation status in both histologies defined as hypomethylated (*n* = 1) or hypermethylated (*n* = 22) in both germinomas/seminomas/dysgerminoma and YST.

### Statistical analysis

Fisher’s exact tests were used to identify significant differences in patient age (0–10, 11–19 years), sex (male, female, gonadal dysgenesis), and tumour location (intracranial, extragonadal, ovary, testes) by histology (germinomas/seminomas/dysgerminoma, mixed, teratoma, YST). Two-sided *p*-values for tests of statistical significance with an alpha of 0.05 were estimated. Patient and tumour characteristic analyses were done in SAS version 9.4 (Cary, North Carolina).

## Results

### Patient and tumour characteristics

There were significant differences in age, sex, and tumour location by GCT histologic subtype (Table 1). When considering age category, 76% of both germinomas/seminoma/dysgerminoma and mixed tumours were diagnosed among children aged 11–19 years, teratomas were equally distributed by age, and 71% of yolk sac tumours (YST) were among children aged 0–10 years (*p* < 0.01). Approximately 60% of germinomas/seminoma/dysgerminoma and teratomas, 32% of mixed tumours and 53% of YST were diagnosed among females (*p* < 0.01). Tumour location varied significantly by GCT histologic subtype (*p* < 0.01) with 58% of germinomas/seminoma/dysgerminoma diagnosed in the ovary (dysgerminoma), 61% of mixed tumours diagnosed in the testes, teratomas were more evenly distributed among tumour locations, and no YSTs were intracranial but were more frequently gonadal in location.

**Table 1:** Patient and germ cell tumour characteristics by histologic subtype

	Germinoma/seminoma /dysgerminoma (*N* = 21)^a^	Mixed/other (*N* = 54)^a^	Teratoma (*N* = 9)^a^	Yolk sac (*N* = 70)^a^	Total (*N* = 154)^a^
Covariate	*n* (%)	*n* (%)	*n* (%)	*n* (%)	*n* (%)
Age					
0– < 11	5 (23.8)	13 (24.1)	5 (55.6)	49 (71.0)	72 (47.1)
11–19	16 (76.2)	41 (75.9)	4 (44.4)	20 (29.0)	81 (52.9)
Missing	0	0	0	1	1
Sex					
Male	7 (33.3)	37 (68.5)	3 (37.5)	29 (47.5)	76 (52.8)
Female	12 (57.1)	17 (31.5)	5 (62.5)	32 (52.5)	66 (45.8)
Gonadal dysgenesis	2 (9.5)	0 (0.0)	0 (0.0)	0 (0.0)	2 (1.4)
Missing	0	0	1	9	10
Tumour location					
Intracranial	3 (15.8)	3 (5.6)	2 (22.2)	0 (0.0)	8 (5.5)
Extragonadal	1 (5.3)	6 (11.1)	4 (44.4)	18 (28.6)	29 (20.0)
Ovary	11 (57.9)	12 (22.2)	3 (33.3)	21 (33.3)	47 (32.4)
Testis	4 (21.1)	33 (61.1)	0 (0.0)	24 (38.1)	61 (42.1)
Missing	2	0	0	7	9

^a^Fisher’s exact *p*-values all < 0.01 for each covariate and histologic subtype

### Histologic subtype clustering independent of age

PCA of the gene methylation beta values for all probes was used to visualise methylation differences by tumour histology, sex (results not shown) and age group. When we evaluated tumour histology, principal component (PC) 1 discriminated between YST and all other histologic subtypes while PC2 separated germinomas/seminomas/dysgerminoma from the mixed tumours and teratoma, which overlapped one another (Fig. 1a). PC1 explained 25.5% of the variance between samples while 12.7% of the variance was explained by PC2. There was no noticeable clustering pattern by age (Fig. 1b).

### Differential methylation by histologic subtype

Comparison of methylation levels across the genome between GCT histologic subtypes identified 8481 differentially methylated regions (DMRs; FWER < 0.05). When stratifying by age group (0–10 vs. 11–19 years), 2228 DMRs (FWER < 0.05) were identified; however, these differences did not persist when tumour histology and age were included as variables in the model used to test for differential methylation indicating that the age-specific DMRs were not preserved between different histologic subtypes.

**Fig. 2:**
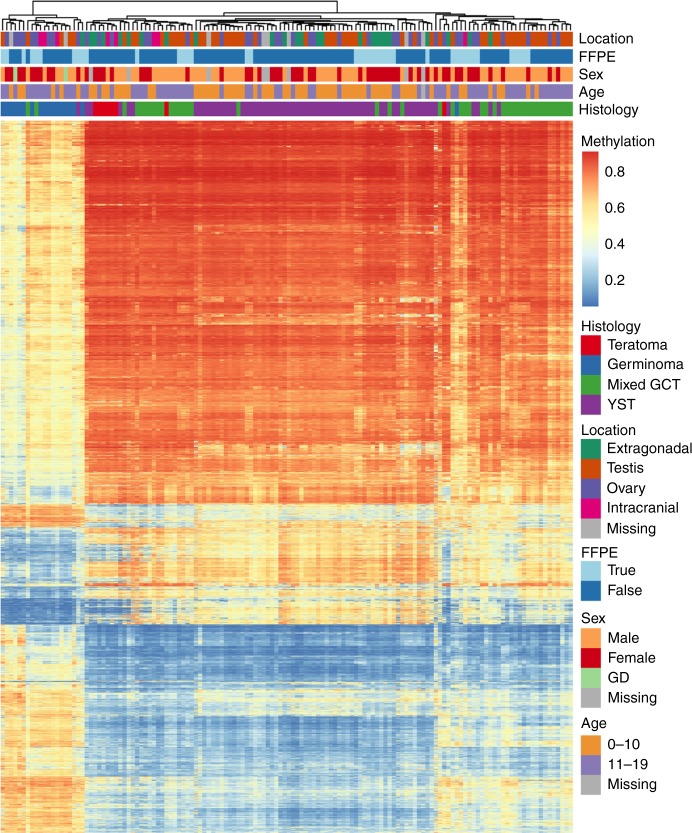
Unsupervised, hierarchical clustering was performed on the average methylation value for all the probes within each DMR for each sample. Colour coding of tumour characteristics is at the top and normalised methylation values are shown in each row from low methylation (blue) to higher methylation (red). Germinoma includes germinoma, seminoma and dysgerminoma

Unsupervised, hierarchal clustering was conducted using the methylation beta values for each probe within the differentially methylated regions (DMRs) identified between the histological subtypes (Fig. 2). Among the pure histologic subtypes 17/18 germinomas/seminoma/dysgerminoma and 52/65 YST consistently clustered together. In general, teratoma and mixed GCTs clustered with their respective subtype, but showed some variability in clustering location. Germinomas/seminoma/dysgerminoma displayed a unique methylation pattern characterised by lower levels of epigenome-wide methylation relative to other tumour types, which were more similar to one another. Unlike histologic subtype, age, sex, tumour location, and FFPE status were not associated with specific clusters.

### Differential methylation between germinomas/seminomas/dysgerminoma and YST

Comparing the pure histologic subtypes of germinomas/seminoma/dysgerminoma and YST, we identified pathways differentially associated with histologic subtype. Of the 8481 DMRs identified between all histologic subtypes, 2969 loci showed increased methylation in germinomas/seminoma/dysgerminoma relative to YST and 5512 loci showed decreased methylation in germinomas/seminoma/dysgerminoma relative to YST.

**Fig. 3:**
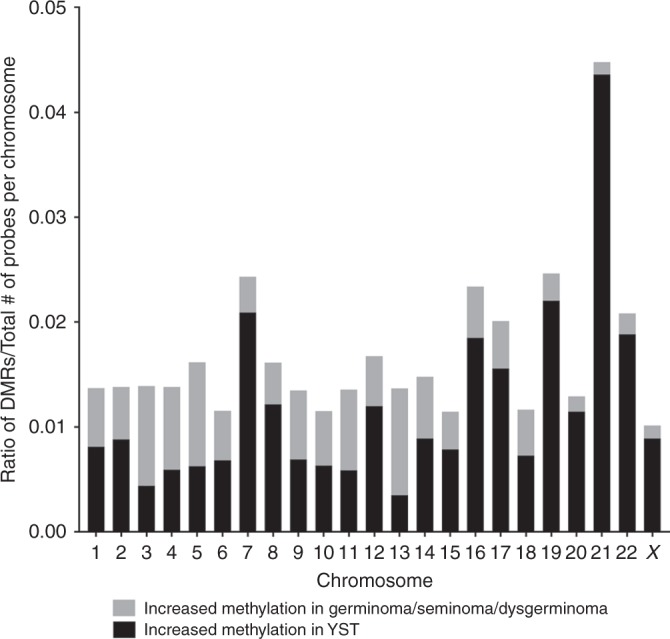
Ratio of DRMs to the total number of probes for DMRs with increased methylation in germinoma relative to YST (grey) and increased in YST relative to germinoma/seminoma/dysgerminoma (black)

We compared the ratio of the number of DMRs with a difference in methylation beta value greater than the absolute value of 0.2 to the total number of probes on each chromosome to determine whether DMRs were evenly distributed across the genome (Fig. 3). The highest ratio of DMRs to total probes were identified on chromosomes 7, 16, 17, 19, 21 and 22, with a particularly notable ratio for increased methylation in YST relative to germinomas/seminomas/dysgerminoma on chromosome 21. Germinomas/seminoma/dysgerminoma had a higher ratio of DMRs to total probes representing increased methylation relative to YST on chromosomes 3, 5 and 13. We observed one gene (ADAP1) that was hypermethylated in both germinomas/seminomas/dysgerminoma and YST (both *β* > 0.7) and 22 genes (results not shown) that were hypomethylated in both germinomas/seminomas/dysgerminoma and YST (both *β* < 0.3); four of these still reached our threshold as DMRs (differential *β* > 0.2), but did not meet the threshold for inclusion in the pathway analysis. The remaining DMRs had average differential beta values of 0.29 (standard deviation = 0.07). Additionally, we identified 51 genes that were hypomethylated (*β* < 0.3) in one histologic subtype and hypermethylated (*β* > 0.7) in the other histologic subtype (Supplemental Table 2).

### Pathway analysis of DMRs between germinomas/seminomas/dysgerminoma and YST

Ingenuity pathway analysis (IPA) was used to identify pathways that were enriched in the top 10% of loci based on the absolute difference in beta values with differential methylation in germinomas/seminoma/dysgerminoma and YST. Compared to YST, among germinomas/seminomas/dysgerminoma there were 346 pathways with decreased methylation and 246 pathways with increased methylation. Pathways with *p*-values < 0.01 are presented in Table 2 (additional pathways in Supplemental Table 1). The top pathways with decreased methylation in germinomas/seminomas/dysgerminoma relative to YST include the TWEAK signalling, tec kinase signalling, and RAR activation pathways. The highly significant pathways with increased methylation in germinomas/seminoma/dysgerminoma relative to YST include the axonal guidance signalling, signalling by rho family GTPases, and the breast cancer regulation by stathmin1 pathways.

**Table 2:** Canonical pathways represented among the top 10% of decreased and increased methylated genes from differentially methylated regions in germinoma relative to yolk sac tumours (*p*-values < 0.01)

IPA pathway	Genes	*p*-value
Decreased methylation in germinoma^a^ compared to YST		
TWEAK signalling	TRAF3, TRAF2, TNFSF12, CASP8, CASP7, TRAF1	0.0001
Cardiac hypertrophy signalling	ADCY3, RHOT2, HAND1, ADRB3, PIK3R3, NKX2–5, PLCD3, CACNA1E, GNAO1, PRKACA, GNB2, IGF1R, PIK3CD, GNB1L, FNBP1	0.0001
Tec kinase signalling	PTK2, PIK3R3, TNFSF12, GNAO1, RHOT2, GNB2, PIK3CD, JAK3, GNB1L, TNF, FNBP1, PRKCZ	0.0001
Sphingosine-1-phosphate signalling	PTK2, PIK3R3, PLCD3, PDGFA, ADCY3, RHOT2, PIK3CD, CASP8, FNBP1, CASP7	0.0002
Cytotoxic T lymphocyte-mediated apoptosis of target cells	CD247, B2M, HLA-A, CASP8, CASP7	0.0004
Role of NFAT in cardiac hypertrophy	PIK3R3, PLCD3, NKX2–5, HDAC7, ADCY3, GNB2, PRKACA, IGF1R, HAND1, PIK3CD, GNB1L, PRKCZ	0.0004
Molecular mechanisms of cancer	RAPGEF1, RHOT2, ADCY3, PRKCZ, PIK3R3, PTK2, NF1, CDK20, GNAO1, E2F1, PRKACA, PIK3CD, CASP8, JAK3, NOTCH1, FNBP1, CASP7, LRP1	0.001
NF-κB signalling	PIK3R3, TRAF3, TRAF2, PRKACA, IGF1R, PIK3CD, LTBR, CASP8, TNFRSF1B, TNF, PRKCZ	0.001
Role of macrophages, fibroblasts and endothelial cells in rheumatoid arthritis	TRAF3, PDGFA, PRKCZ, PIK3R3, PLCD3, TRAF2, APC2, GNAO1, PIK3CD, LTBR, SOST, TNFRSF1B, TNF, LRP1, TRAF1	0.001
RAR activation	PIK3R3, PRMT2, CSK, NR2F2, ADCY3, PRKACA, PIK3CD, NCOR2, PML, PRKCZ, SMARCA4	0.001
Huntington’s disease signalling	SDHA, PRKCZ, PIK3R3, DNAJC5, HDAC7, GNB2, IGF1R, VAMP3, PIK3CD, NCOR2, CASP8, GNB1L, CASP7	0.001
Small-cell lung cancer signalling	PTK2, PIK3R3, TRAF3, TRAF2, E2F1, PIK3CD, TRAF1	0.001
Type I diabetes mellitus signalling	CD247, CD28, TRAF2, HLA-A, GAD1, CASP8, TNFRSF1B, TNF	0.002
CXCR4 signalling	PTK2, PIK3R3, GNAO1, ADCY3, RHOT2, GNB2, PIK3CD, GNB1L, FNBP1, PRKCZ	0.002
Lymphotoxin β-receptor signalling	PIK3R3, TRAF3, TRAF2, PIK3CD, LTBR, TRAF1	0.002
Death receptor signalling	TRAF2, TNFSF12, LMNA, CASP8, TNFRSF1B, TNF, CASP7	0.002
Thrombin signalling	PTK2, PIK3R3, PLCD3, GNAO1, ADCY3, RHOT2, GNB2, PIK3CD, GNB1L, FNBP1, PRKCZ	0.002
Myc-mediated apoptosis signalling	PIK3R3, YWHAG, IGF1R, PIK3CD, CASP8, PRKCZ	0.002
TNFR2 signalling	TRAF2, TNFRSF1B, TNF, TRAF1	0.003
Colorectal cancer metastasis signalling	PIK3R3, GRK2, ADCY3, RHOT2, GNB2, PRKACA, PIK3CD, JAK3, GNB1L, TNF, FNBP1, LRP1	0.004
CREB signalling in neurons	PIK3R3, PLCD3, GRM3, GNAO1, ADCY3, GNB2, PRKACA, PIK3CD, GNB1L, PRKCZ	0.004
Relaxin signalling	PIK3R3, PDE9A, GNAO1, ADCY3, GNB2, PRKACA, PIK3CD, GNB1L, PRKCZ	0.004
Gαq signalling	PIK3R3, GRK2, CSK, RHOT2, GNB2, PIK3CD, GNB1L, FNBP1, PRKCZ	0.004
CD40 signalling	PIK3R3, TRAF3, TRAF2, PIK3CD, JAK3, TRAF1	0.004
Glioblastoma multiforme signalling	PIK3R3, PLCD3, NF1, PDGFA, E2F1, RHOT2, IGF1R, PIK3CD, FNBP1	0.005
IGF-1 signalling	PTK2, PIK3R3, YWHAG, PRKACA, IGF1R, PIK3CD, PRKCZ	0.005
P2Y purigenic receptor signalling pathway	PIK3R3, PLCD3, ADCY3, GNB2, PRKACA, PIK3CD, GNB1L, PRKCZ	0.005
Antigen presentation pathway	B2M, PSMB9, HLA-A, TAP1	0.006
Cardiac β-adrenergic Signalling	CACNA1E, GRK2, PDE9A, PPP1R3C, ADCY3, GNB2, PRKACA, GNB1L	0.006
Induction of apoptosis by HIV1	TRAF2, CASP8, TNFRSF1B, TNF, TRAF1	0.007
Role of PKR in interferon induction and antiviral response	TRAF3, TRAF2, CASP8, TNF	0.008
Increased methylation in germinoma relative to YST		
Axonal guidance signalling	NTNG1, EFNB2, SRGAP3, BMP4, GLIS1, ARHGEF7, GNA12, C9orf3, ADAM19, PFN2, HHIP, KLB, GNG7, PRKCB	0.0005
Signalling by Rho family GTPases	MAP3K9, CDH1, GNA12, ARHGEF7, PIP4K2B, ARHGEF18, ARHGEF3, KLB, GNG7	0.002
Breast cancer regulation by stathmin1	CAMK1D, ARHGEF7, ARHGEF18, ITPR1, ARHGEF3, KLB, GNG7, PRKCB	0.002
RhoGDI signalling	CDH1, GNA12, ARHGEF7, PIP4K2B, ARHGEF18, ARHGEF3, GNG7	0.003
TR/RXR activation	F10, PDE3B, PFKP, THRB, KLB	0.004
Coagulation system	F10, F3, SERPINF2	0.006
Thrombin signalling	CAMK1D, GNA12, ITPR1, ARHGEF3, KLB, GNG7, PRKCB	0.007
Guanosine nucleotides degradation III	NT5C2, ACPP	0.007
Relaxin signalling	PDE3B, GNA12, PDE4D, NPR2, KLB, GNG7	0.007
Urate biosynthesis/inosine 5’-phosphate degradation	NT5C2, ACPP	0.009

^a^Germinoma includes germinoma, seminoma and dysgerminoma

## Discussion

In our histologically diverse epigenome-wide study of 154 paediatric GCTs, we identified 8481 regions with differential methylation by histologic subtype, which is in-line with the number of DMRs identified in a study of paediatric neuroblastoma samples.^33^ When overall methylation levels were summarised via PCA, germinomas/seminoma/dysgerminoma and YST were clustered tightly into two distinct groups and were the most distant from each other along the axis representing principal component 1. Age, sex, location and FFPE status did not create distinct groups when visualising the first two principal components. In unsupervised, hierarchical clustering, germinomas/seminoma/dysgerminoma formed a strong cluster while the remaining tumour types showed more variability in clustering by histology. Importantly, these clustering patterns did not appear to be driven by FFPE status, sex, age and tumour location. Germinomas/seminoma/dysgerminoma displayed a strong tendency toward epigenome-wide hypomethylation relative to the other histologic subtypes. Finally, when comparing methylation profiles of germinomas/seminoma/dysgerminoma and YST, we observed increased methylation among YSTs particularly in established tumour suppressor genes and immune regulatory, cell proliferation and angiogenesis pathways.

While there is considerable variation in age at diagnosis by GCT histologic subtype, in our analysis of paediatric and adolescent GCTs, histology rather than age was the major source of variation across the samples as visualised by the first two principal components. This is consistent with previous analyses of methylation in intracranial GCTs^23^ and gene expression in paediatric GCTs^34^ where molecular differences were predicted more accurately by histology than patient age. We observed consistent clustering of germinomas/seminoma/dysgerminoma cases based on methylation beta values and this subtype displayed lower levels of methylation relative to other subtypes, which was similarly reported among intracranial GCTs.^23^ Conversely, we and others have reported that YST and teratomas generally clustered together and displayed high global methylation relative to the other histologic subtypes.^22^ While there are few studies on differential methylation by histologic subtype in paediatric GCTs, studies of adult testicular GCT have reported similar differences in methylation patterns between seminoma and nonseminoma tumour subtypes.^22,35^

There are established differences in response to treatment by histologic subtype, with nonseminomas, such as YST, exhibiting poorer responses to radio- and chemotherapy.^36^ Therefore, characterising differentially methylated genes and pathways between YST and germinomas/seminomas/dysgerminoma may aid in understanding therapy resistance and identify potentially druggable targets. Studies of paediatric GCTs have reported that Wnt pathway regulator,^37^ the adenomatous polyposis coli (APC) gene, was methylated in YST but not germinoma^19,38,39^ leading to activation of Wnt signalling.^40,41^ In our study, we observed hypermethylation of Wnt pathway regulator, APC2, in YST relative to germinomas/seminoma/dysgerminoma (results not shown). We observed increased methylation for ASCL2, a Wnt target gene,^42^ and NPY, an activator of Wnt pathway proteins,^43^ in YST relative to germinomas/seminoma/dysgerminoma as others have reported (results not shown).^19^ Additionally, in YST we observed hypermethylation of previously identified genes suspected to be tumour suppressors including: HOXA9, SCGB3A1, IRF5, CASP8 and LTB4R.^19,20^ In agreement with Amatruda et al.^20^ who reported differential methylation between YST compared to the other histologic subtypes, we observed differential methylation for YST and germinomas/seminoma/dysgerminoma for RASSF1, SCGB3A1, HOXA9, FGF3, PDGFRB, NPY, ASCL2, CDK10 and GUCY2D. In a previous analysis, Noor et al.^44^ identified 72 genes with differential methylation and correlated gene expression in seminoma and YST from adult TGCT cell lines and paediatric GCT tumour samples.^34^ In our analysis, we observed differential methylation in 15/72 genes identified by Noor et al. This suggests that there may be a number of important genes that are dysregulated in both paediatric and adult YSTs relative to seminomas. Collectively, these findings may highlight potential target genes and pathways for drug development to improve outcomes for YST in particular.

When we used IPA to identify pathways over-represented from the identified DMRs in germinomas/seminomas/dysgerminoma compared to YST, we identified pathways with both decreased and increased methylation. Pathways with significantly decreased methylation in germinomas/seminoma/dysgerminoma relative to YST include the TWEAK, tec kinase signalling, RAR activation and a number of immune cell-related pathways. The TWEAK signalling pathway is hypothesised to contribute to tumour proliferation and angiogenesis^45^ while the tec kinase signalling pathway plays a role in the activation of T- and B-cells.^46,47^ The RAR activation pathway plays an important role in developmental differentiation of cells.^48^ Various immune cell-related pathways had decreased methylation in germinomas/seminoma/dysgerminoma vs. YST. While a smaller number of pathways had increased methylation in germinomas/seminoma/dysgerminoma compared to YST, these pathways did not appear to be related to immune signalling or immune cell development as observed for the pathways with decreased methylation in germinomas/seminoma/dysgerminoma relative to YST. Identified pathways with increased methylation in germinomas/seminoma/dysgerminoma include the axonal guidance signalling, signalling by rho family GTPases, and the breast cancer regulation by stathmin1 pathways. The role of these specific pathways in the development of one histologic subtype of GCT over another remains unclear, but they have been associated with tumour development by contributing to cell proliferation, cell migration and angiogenesis.^49–51^

We identified a number of chromosomes with a higher ratio of DMRs to total probes in YST compared to germinomas/seminoma/dysgerminoma, particularly for chromosomes 7, 16, 17, 19, 21 and 22. In the literature, there are reports of copy number alterations and gains of chromosomes in GCTs, which may explain the increased methylation we observed in YST compared to germinomas/seminoma/dysgerminoma. There is a reported gain of 17q in embryonic cell lines^52^ and testicular nonseminoma.^53^ Gains of chromosomes 7, 8, 17, 21, 22 in testicular germ cell tumours (TGCTs) and GCT case reports have also been described.^53–56^ Few studies have reported a gain in chromosome 19,^56^ where we observed many DMRs with increased methylation in YST vs. germinomas/seminoma/dysgerminoma. We observed the largest ratio of DMRs to total probes with increased methylation in YST relative to germinomas/seminoma/dysgerminoma for chromosome 21. There are reports of an increased risk of GCTs, particularly testicular tumours, among males with Down’s syndrome.^57–59^ When we identified genes that were hypomethylated (*β* < 0.3) in one histologic subtype and hypermethylated (*β* > 0.7) in another histologic subtype, in contrast to our first analysis using an absolute value of 0.2 as a differential methylation cut-off, we found a smaller number of genes that were hypomethylated in one histologic subtype yet hypermethylated in the other. These findings highlight regions of the genome that may be important in GCT development.

Our findings should be viewed in light of the following limitations. While we present an epigenome-wide study of methylation from a large number of paediatric GCTs, we were unable to subset the mixed tumours and immature and mature teratomas based on their histologic components, as this information was not available for our cases. Similarly, we were restricted by sample size to examine germinoma, seminoma and dysgerminoma separately. We were unable to examine methylation profiles in association with treatment response or survival, as we did not have adequate outcome data for the participants. We did not have normal tissue samples available to allow comparison of normal and tumour tissue methylation. Finally, in the present study, we do not have gene expression data available to examine the correlation between methylation and expression for the genes of interest; therefore, while gene methylation often correlates with gene expression,^60,61^ exploring these relationships within histologic subtypes of paediatric GCTs remains to be completed. Interestingly, Amatruda and colleagues^20^ found negative correlations between gene methylation and expression for some genes among teratoma samples suggesting a mechanistic role for methylation in gene expression control in paediatric GCTs. Further, gene methylation may also be informative beyond the regulation of gene expression as it may also serve as a marker of exposure or outcome as discussed by Michels et al.^62^

In conclusion, we observed distinct differences in gene-specific methylation between the histologic subtypes of GCT, particularly between germinoma/seminoma/dysgerminoma and YST. While it has been hypothesised that GCTs arise from the PGC and the resulting tumour type depends on the maturity of the PGC,^63^ environmental exposures that impact tumour microenvironment may exert phenotype modifying pressures that encourage tumours in the gonads or extragonadal locations to develop into one histologic subtype over another under the control of DNA methylation. The data from our epigenome-wide study along with the findings of other research groups suggest that alteration of methylation patterns could be a primary mechanism of histologic subtype determination. We and others^19,20^ have observed hypermethylation of tumour suppressor genes in YST, suggesting a potential mechanism for tumour initiation and chemotherapeutic resistance. Future work investigating these methylation changes in histologic subtypes of GCTs compared to methylation in PGCs and other precursor cell types may be informative in understanding GCT aetiology and the timing of GCT initiation. Further, the identification of windows of susceptibility during the prenatal and early developmental period may shed light on exposures that impact DNA methylation and lead to risk reduction strategies.

## Electronic supplementary material

Supplemental Table 1

Supplemental Table 2

## **Availability of data and materials:**

Data may be made available upon a reasonable request to the corresponding author.
