# Supplementary material for: Differences in DNA methylation profiles by histologic subtype of paediatric germ cell tumours: a report from the Children’s Oncology Group
Source: Br J Cancer. 2018 Oct 5;119(7):864–72. doi: 10.1038/s41416-018-0277-5 (PMC6189207; doi:10.1038/s41416-018-0277-5)
Supplement: Supplementary file 1 — Supplemental Table 1 [file 41416_2018_277_MOESM1_ESM.docx]

**Supplemental Table 1. Additional canonical pathways represented among the top 10% of down and up regulated genes from differentially methylated regions in germinoma* relative to yolk sac tumors**

| IPA Pathway | Genes | p-value | |
| --- | --- | --- | --- |
| Decreased methylation in germinoma* compared to YST | | |  |
| Ceramide Signaling | PIK3R3, PIK3CD, TNFRSF1B, TNF, ENPP7, PRKCZ | 0.01 | |
| Renin-Angiotensin Signaling | PTK2, PIK3R3, ADCY3, PRKACA, PIK3CD, TNF, PRKCZ | 0.01 | |
| IL-9 Signaling | PIK3R3, PIK3CD, JAK3, TNF | 0.01 | |
| Glioma Invasiveness Signaling | PTK2, PIK3R3, RHOT2, PIK3CD, FNBP1 | 0.01 | |
| TR/RXR Activation | PIK3R3, SLC16A3, PIK3CD, TBL1XR1, PFKP, NCOR2 | 0.01 | |
| Type II Diabetes Mellitus Signaling | PIK3R3, TRAF2, PIK3CD, TNFRSF1B, TNF, ENPP7, PRKCZ | 0.01 | |
| CTLA4 Signaling in Cytotoxic T Lymphocytes | CD247, B2M, PIK3R3, CD28, HLA-A, PIK3CD | 0.01 | |
| Dendritic Cell Maturation | B2M, PIK3R3, PLCD3, HLA-A, PIK3CD, LTBR, COL18A1, TNFRSF1B, TNF | 0.01 | |
| Ephrin B Signaling | PTK2, NCK2, GNAO1, GNB2, GNB1L | 0.01 | |
| Production of Nitric Oxide and Reactive Oxygen Species in Macrophages | PIK3R3, PPP1R3C, RHOT2, PIK3CD, JAK3, TNFRSF1B, TNF, FNBP1, PRKCZ | 0.01 | |
| PAK Signaling | PTK2, NCK2, PIK3R3, PDGFA, PIK3CD, TNF | 0.01 | |
| 14-3-3-mediated Signaling | PIK3R3, PLCD3, TRAF2, YWHAG, PIK3CD, TNF, PRKCZ | 0.01 | |
| TNFR1 Signaling | TRAF2, CASP8, TNF, CASP7 | 0.02 | |
| IL-15 Signaling | PTK2, PIK3R3, PIK3CD, JAK3, TNF | 0.02 | |
| IL-15 Production | PTK2, JAK3, PRKCZ | 0.02 | |
| Th1 Pathway | CD247, PIK3R3, CD28, HLA-A, PIK3CD, JAK3, NOTCH1 | 0.02 | |
| Breast Cancer Regulation by Stathmin1 | PIK3R3, PPP1R3C, E2F1, ADCY3, GNB2, PRKACA, PIK3CD, GNB1L, PRKCZ | 0.02 | |
| Semaphorin Signaling in Neurons | PTK2, PLXNA1, RHOT2, FNBP1 | 0.02 | |
| G-Protein Coupled Receptor Signaling | PIK3R3, OPRD1, GRK2, GRM3, PDE9A, GNAO1, ADCY3, PRKACA, PIK3CD, ADORA2A, ADRB3 | 0.02 | |
| Insulin Receptor Signaling | PIK3R3, RAPGEF1, PPP1R3C, PRKACA, PIK3CD, PRKCZ, PTPRF | 0.02 | |
| Gαs Signaling | ADCY3, GNB2, PRKACA, GNB1L, ADORA2A, ADRB3 | 0.02 | |
| Transcriptional Regulatory Network in Embryonic Stem Cells | HAND1, OTX1, ONECUT1, GSX2 | 0.02 | |
| Amyotrophic Lateral Sclerosis Signaling | PRPH, PIK3R3, CACNA1E, HECW1, PIK3CD, CASP7 | 0.02 | |
| Nitric Oxide Signaling in the Cardiovascular System | PIK3R3, CACNA1E, PRKACA, PIK3CD, PRKCZ, ADRB3 | 0.02 | |
| Glioma Signaling | PIK3R3, PDGFA, E2F1, IGF1R, PIK3CD, PRKCZ | 0.02 | |
| Neuropathic Pain Signaling In Dorsal Horn Neurons | PIK3R3, PLCD3, GRM3, PRKACA, PIK3CD, PRKCZ | 0.03 | |
| Growth Hormone Signaling | PIK3R3, IGF1R, PIK3CD, PRKCZ, ONECUT1 | 0.03 | |
| AMPK Signaling | PIK3R3, PRKACA, CHRNE, PIK3CD, PFKL, PFKP, GNB1L, SMARCA4, ADRB3 | 0.03 | |
| Leptin Signaling in Obesity | PIK3R3, PLCD3, ADCY3, PRKACA, PIK3CD | 0.03 | |
| Signaling by Rho Family GTPases | PTK2, PIK3R3, GNAO1, RHOT2, GNB2, PIK3CD, GNB1L, FNBP1, PRKCZ, PKN1 | 0.03 | |
| α-Adrenergic Signaling | ADCY3, GNB2, PRKACA, GNB1L, PRKCZ | 0.03 | |
| Th2 Pathway | CD247, PIK3R3, CD28, HLA-A, PIK3CD, JAK3, NOTCH1 | 0.03 | |
| G Beta Gamma Signaling | GNAO1, GNB2, PRKACA, GNB1L, PRKCZ | 0.03 | |
| Role of NFAT in Regulation of the Immune Response | CD247, PIK3R3, CD28, HLA-A, GNAO1, GNB2, PIK3CD, GNB1L | 0.03 | |
| Crosstalk between Dendritic Cells and Natural Killer Cells | CD28, HLA-A, LTBR, TNFRSF1B, TNF | 0.03 | |
| Gαi Signaling | OPRD1, GRM3, ADCY3, GNB2, PRKACA, GNB1L | 0.03 | |
| Apoptosis Signaling | LMNA, CASP8, TNFRSF1B, TNF, CASP7 | 0.03 | |
| OX40 Signaling Pathway | CD247, B2M, TRAF3, TRAF2, HLA-A | 0.03 | |
| Endothelin-1 Signaling | PIK3R3, PLCD3, GNAO1, ADCY3, PIK3CD, CASP8, CASP7, PRKCZ | 0.03 | |
| iCOS-iCOSL Signaling in T Helper Cells | CD247, PIK3R3, CD28, HLA-A, CSK, PIK3CD | 0.03 | |
| IL-1 Signaling | GNAO1, ADCY3, GNB2, PRKACA, GNB1L | 0.03 | |
| cAMP-mediated signaling | OPRD1, GRK2, GRM3, PDE9A, GNAO1, ADCY3, PRKACA, ADORA2A, ADRB3 | 0.03 | |
| Hepatic Cholestasis | TRAF2, NR1I2, ADCY3, PRKACA, TNFRSF1B, TNF, PRKCZ | 0.04 | |
| PPAR Signaling | TRAF2, PDGFA, NCOR2, TNFRSF1B, TNF | 0.04 | |
| Sumoylation Pathway | RHOT2, PML, ZNF217, FNBP1, UBE2I | 0.04 | |
| UVB-Induced MAPK Signaling | PIK3R3, PIK3CD, HIST3H3, PRKCZ | 0.04 | |
| Role of Osteoblasts, Osteoclasts and Chondrocytes in Rheumatoid Arthritis | PIK3R3, TRAF2, APC2, PIK3CD, ACP5, SOST, TNFRSF1B, TNF ,LRP1 | 0.04 | |
| IL-8 Signaling | PTK2, PIK3R3, RHOT2, GNB2, PIK3CD, GNB1L, FNBP1, PRKCZ | 0.04 | |
| ILK Signaling | PTK2, NCK2, PIK3R3, RHOT2, PIK3CD, MYH7, TNF, FNBP1 | 0.04 | |
| April Mediated Signaling | TRAF3, TRAF2, TRAF1 | 0.04 | |
| Superpathway of Inositol Phosphate Compounds | PIK3R3, PLCD3, CD28, TNS3, DUSP27, PPM1F, PIK3CD, ACP5, PTPRF | 0.04 | |
| Dopamine-DARPP32 Feedback in cAMP Signaling | PLCD3, KCNJ4, CACNA1E, PPP1R3C, ADCY3, PRKACA, PRKCZ | 0.04 | |
| Phagosome Formation | PIK3R3, PLCD3, RHOT2, PIK3CD, FNBP1, PRKCZ | 0.04 | |
| ErbB Signaling | NCK2, PIK3R3, NRG2, PIK3CD, PRKCZ | 0.04 | |
| 3-phosphoinositide Biosynthesis | PIK3R3, CD28, TNS3, DUSP27, PPM1F, PIK3CD, ACP5, PTPRF | 0.04 | |
| ERK/MAPK Signaling | PTK2, PIK3R3, RAPGEF1, YWHAG, PPP1R3C, PRKACA, PIK3CD, HIST3H3 | 0.04 | |
| CD28 Signaling in T Helper Cells | CD247, PIK3R3, CD28, HLA-A, CSK, PIK3CD | 0.04 | |
| Increased methylation in germinoma* relative to YST | | |  |
| Molecular Mechanisms of Cancer | CDH1, BMP4, GNA12, ARHGEF7, CDK6, ARHGEF18, ARHGEF3, KLB, CTNND1, PRKCB | 0.01 | |
| Heparan Sulfate Biosynthesis | HS3ST3B1, XYLT1, EXT1, NDST1 | 0.01 | |
| Extrinsic Prothrombin Activation Pathway | F10, F3 | 0.01 | |
| Adenosine Nucleotides Degradation II | NT5C2, ACPP | 0.01 | |
| GPCR-Mediated Nutrient Sensing in Enteroendocrine Cells | ITPR1, RAPGEF4, GNG7, PRKCB | 0.01 | |
| Phospholipase C Signaling | AHNAK, ARHGEF7, ARHGEF18, ITPR1, ARHGEF3, GNG7, PRKCB | 0.02 | |
| Purine Nucleotides Degradation II (Aerobic) | NT5C2, ACPP | 0.02 | |
| Role of NFAT in Cardiac Hypertrophy | NKX2-5, CAMK1D, ITPR1, KLB, GNG7, PRKCB | 0.02 | |
| Cardiomyocyte Differentiation via BMP Receptors | NKX2-5, BMP4 | 0.02 | |
| IL-8 Signaling | CDH1, ANGPT2, GNA12, KLB, GNG7, PRKCB | 0.02 | |
| Chondroitin Sulfate Biosynthesis | HS3ST3B1, XYLT1, NDST1 | 0.02 | |
| Glutamate Receptor Signaling | SLC17A1, SLC1A7, GNG7 | 0.02 | |
| Dermatan Sulfate Biosynthesis | HS3ST3B1, XYLT1, NDST1 | 0.03 | |
| SAPK/JNK Signaling | MAP3K9, GNA12, KLB, GNG7 | 0.03 | |
| Protein Kinase A Signaling | AKAP2, FLNB, RHO, PDE3B, PDE4D, DUSP15, ITPR1, GNG7, PRKCB | 0.03 | |
| NAD Salvage Pathway II | NT5C2, ACPP | 0.03 | |
| Thrombopoietin Signaling | MPL, KLB, PRKCB | 0.03 | |
| Pyridoxal 5'-phosphate Salvage Pathway | MAP3K9, CDK6, GRK5 | 0.03 | |
| CXCR4 Signaling | GNA12, ITPR1, KLB, GNG7, PRKCB | 0.03 | |
| Nitric Oxide Signaling in the Cardiovascular System | PDE3B, ITPR1, KLB, PRKCB | 0.03 | |
| Glioma Signaling | CAMK1D, CDK6, KLB, PRKCB | 0.04 | |
| Neuropathic Pain Signaling In Dorsal Horn Neurons | CAMK1D, ITPR1, KLB, PRKCB | 0.04 | |
| HGF Signaling | ETS1, MAP3K9, KLB, PRKCB | 0.04 | |
| Remodeling of Epithelial Adherens Junctions | CDH1, EXOC2, CTNND1 | 0.04 | |
| Xenobiotic Metabolism Signaling | MAP3K9, HS3ST3B1, CAMK1D, NDST1, KLB, SOD3, PRKCB | 0.04 | |
| Sonic Hedgehog Signaling | GLIS1, HHIP | 0.04 | |
| Basal Cell Carcinoma Signaling | BMP4, GLIS1, HHIP | 0.04 | |

*Germinoma includes seminomas and dysgerminoma
