# Supplementary material for: Differences in DNA methylation profiles by histologic subtype of paediatric germ cell tumours: a report from the Children’s Oncology Group
Source: Br J Cancer. 2018 Oct 5;119(7):864–72. doi: 10.1038/s41416-018-0277-5 (PMC6189207; doi:10.1038/s41416-018-0277-5)
Supplement: Supplementary file 2 — Supplemental Table 2 [file 41416_2018_277_MOESM2_ESM.docx]

**Supplemental Table 2. Differential beta values for genes with opposite methylation status in germinomas/seminoma/dysgerminoma and yolk sac tumors**

| Gene | Chromosome | Differential β _germinoma*-YST_ |
| --- | --- | --- |
| Genes unmethylated in germinoma* (β<0.3), methylated in YST (β>0.7) | | |
| ARID3A | 19 | -0.448 |
| CASP8 | 2 | -0.423 |
| LINC00936 | 12 | -0.435 |
| LOC145845 | 15 | -0.445 |
| NR2F2 | 15 | -0.500 |
| OTX1 | 2 | -0.521 |
| USP18 | 22 | -0.439 |
| Genes methylated in germinoma* (β>0.7), unmethylated in YST (β<0.3) | | |
| ALPK2 | 18 | 0.418 |
| ALPK2 | 18 | 0.451 |
| APBB1IP | 10 | 0.428 |
| APLP2 | 11 | 0.443 |
| ATG7 | 3 | 0.438 |
| ATG7 | 3 | 0.476 |
| CMSS1 | 3 | 0.497 |
| CSRNP1 | 3 | 0.454 |
| DIXDC1 | 11 | 0.451 |
| DUSP15 | 20 | 0.420 |
| EXOC2 | 6 | 0.439 |
| FEZ1 | 11 | 0.452 |
| FILIP1L | 3 | 0.497 |
| GNLY | 2 | 0.431 |
| JAKMIP2 | 5 | 0.463 |
| JAKMIP2-AS1 | 5 | 0.463 |
| KIRREL3 | 11 | 0.429 |
| KLB | 4 | 0.432 |
| KRTAP3-1 | 17 | 0.444 |
| LINC00676 | 13 | 0.448 |
| LINC01091 | 4 | 0.486 |
| LOC101929544 | 5 | 0.428 |
| LOC102723471 | 17 | 0.524 |
| LOC102724050 | 12 | 0.440 |
| LOC105376365 | 10 | 0.485 |
| MIR1273H | 4 | 0.432 |
| MIR548G | 3 | 0.497 |
| MPL | 1 | 0.458 |
| NAV1 | 1 | 0.472 |
| NEBL-AS1 | 10 | 0.449 |
| NTNG1 | 1 | 0.454 |
| NUMA1 | 11 | 0.418 |
| PARP11 | 12 | 0.486 |
| PDZD3 | 11 | 0.458 |
| PRICKLE1 | 12 | 0.476 |
| PRRT3-AS1 | 3 | 0.451 |
| PTCSC3 | 14 | 0.448 |
| RFC3 | 13 | 0.496 |
| RGMB | 5 | 0.441 |
| RORA | 15 | 0.497 |
| RPEL1 | 10 | 0.518 |
| SERPINF2 | 17 | 0.492 |
| SMIM23 | 5 | 0.427 |
| SRGAP3 | 3 | 0.477 |
| STT3B | 3 | 0.416 |
| USP2 | 11 | 0.449 |
| ZNF385A | 12 | 0.440 |

*Germinoma includes seminomas and dysgerminoma
